# Örjan Ouchterlony and the antigen–antibody double diffusion‐in‐gel: a survey

**DOI:** 10.1111/apm.13480

**Published:** 2024-10-16

**Authors:** Niels Høiby

**Affiliations:** ^1^ Department of Clinical Microbiology Rigshospitalet Copenhagen Denmark; ^2^ Institute of Immunology and Microbiology University of Copenhagen Copenhagen Denmark

**Keywords:** Diffusion‐in‐gel, immunoelectrophoresis, Ouchterlony

## Abstract

The Swedish scientist Örjan Ouchterlony published four ground‐breaking papers 1948–1966 in Acta Pathol Microbiol Scand where he described a new method of antigen–antibody reactions in gel. He described and defined the ‘reaction of identity’ and ‘reaction of partial identity’ when he used related antigens and ‘reaction of non‐identity’ when he used non‐related antigens. His results inspired scientists in other countries to further develop and modify the ‘Ouchterlony method’ which became useful for both scientific and clinical purposes. This survey describes how the methods were discovered and how they became modified and improved and how they were used, but also underlines that the original Ouchterlony method is still used.

## INTRODUCTION

Ørjan Thomas Ouchterlony MD (1914–2004) was a Swedish bacteriologist and Immunologist who was educated and trained in Stockholm at the Karolinska Institute and the Swedish State Bacteriology Laboratory 1935–1952 and became professor of bacteriology at the medical faculty in Gothenburg 1952–1980. He published four papers of ground‐breaking immunological experiments in *Acta Pathol Microbiol Scand* in the period 1949–1966 which started a new area of research in many countries.

## OUCHTERLONY'S WORK ON ANTIGEN–ANTIBODY REACTIONS IN GEL

At the Swedish State Bacteriology Laboratory, he worked on his doctoral thesis (1) which focused on improving the laboratory diagnosis of the *Corynebacterium diphtheriae* strains which produce the diphtheria toxin. At that time the routine method for detecting the toxin was either to inject a fluid culture of *C. diphtheriae* into guinea pigs or a flocculation test in fluid media between the toxin and antibodies from immunized horses (2).

**Fig. 1:**
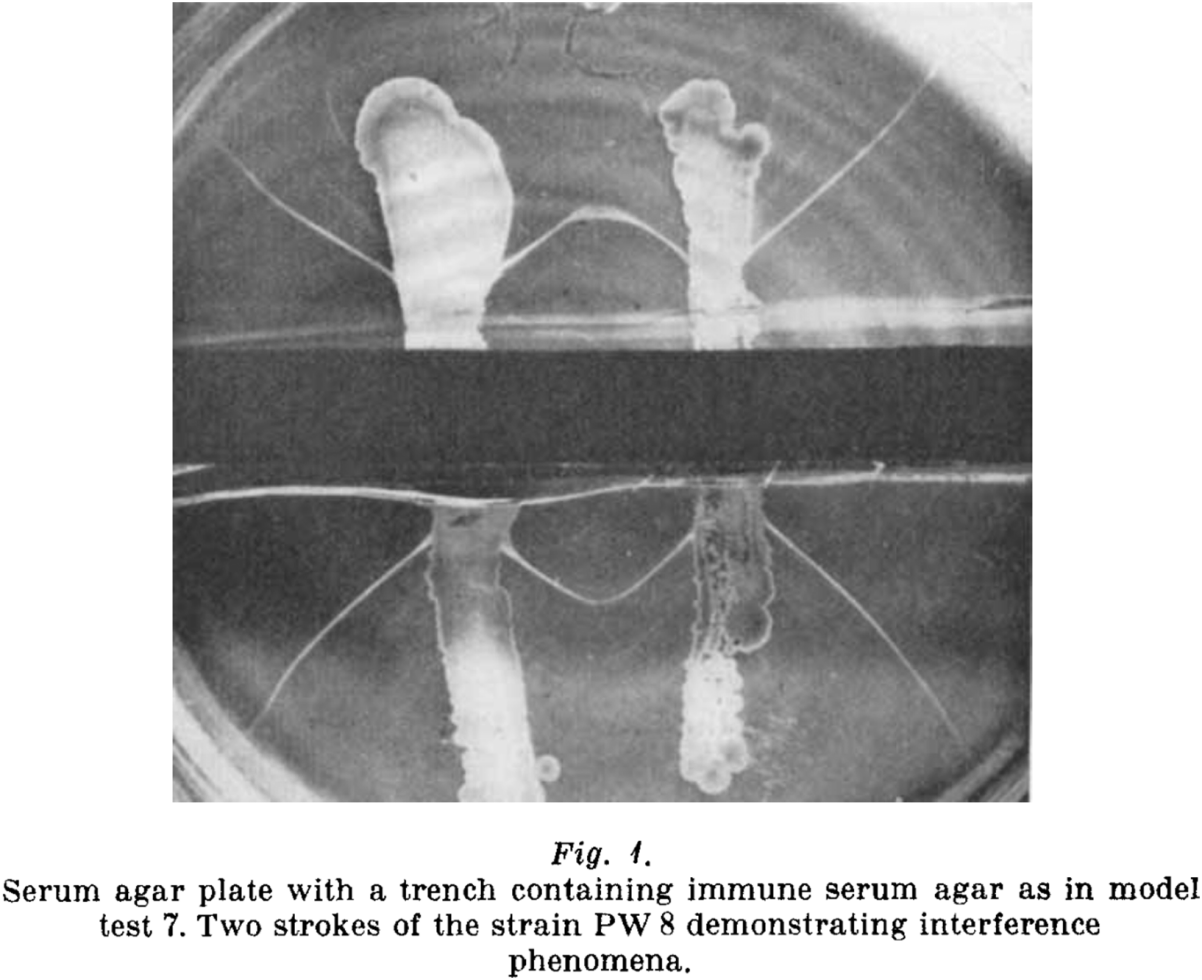
The original Figure in Reference (3) showing the immune precipitation‐in‐gel between anti‐diphtheria antibodies in the horizontal trench and the diphtheria toxin diffusing from the four vertical streaks of a toxin producing *C. diphtheria* strain PW8. A ‘reaction of identity’ (complete fusion) is seen between the precipitates from the right and left streaks of bacterial colonies.

Ouchterlony published his results in 1948 (3), and the figures show sharp precipitation lines and ‘reaction of identity’ (Fig. 1) between the lines from toxins diffusing from closely located bacterial colonies. The precipitation lines, however, did not become visible until after 48–96 h. In the next article published the same year, he elaborated on the method and compared it with the flocculation method (4). He showed that the limit of sensitivity was about one FIU/mL (Flocculation International Unit) (4). This was a completely new qualitative, sensitive, and reproducible test to detect toxin producing strains of *C. diphtheria*, and the method is still used for many other immunological purposes. Today, the presence of the gene coding for the toxin in *C. diphtheria* is detected by a PCR method, but the golden standard showing that the strain is actually producing the toxin is still a variant of the Ouchterlony test (5, 6).

**Fig. 2:**
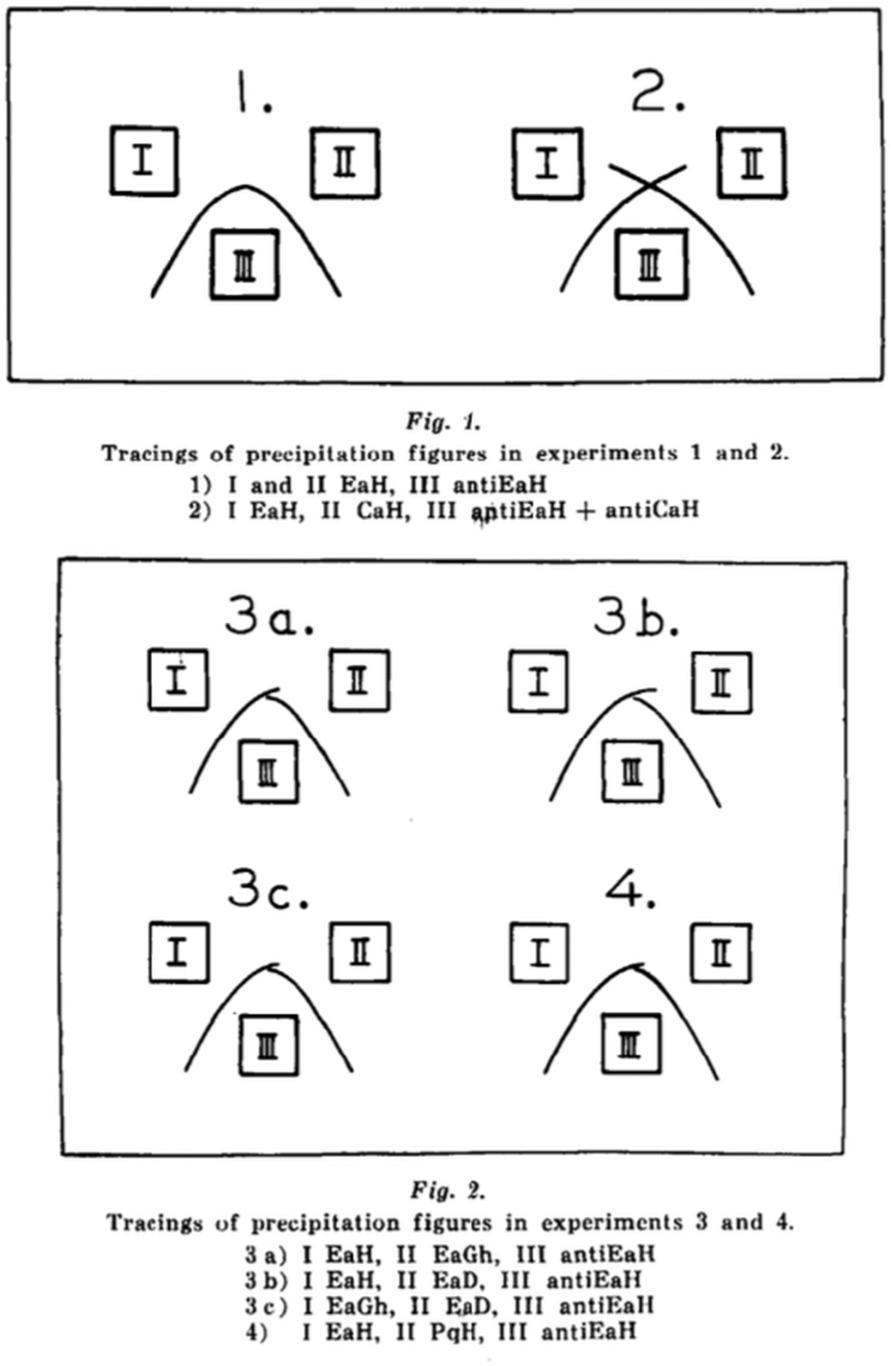
Top is the original Fig. 1 in Reference (7). EaH, egg albumin from hen; CaH, conalbumin (= egg transferrin) from hen, AntiEaH and AntiCaH are immune serum (from immunized rabbits) against EaH and CaH. *Experiment 1* shows fused precipitation lines = ‘reaction of identity’. *Experiment 2* shows crossing precipitation lines without interference = ‘reaction of non‐identity’. Bottom is the original Fig. 2 in Reference (7). Abbreviations as above, with addition of EaGh, egg albumin from the zoologically related guinea hen; EaD, egg albumin from the zoologically less related duck; PqH, egg albumin from hen treated with enzyme from *Bacillus subtillis*. *Experiments 3a, 3b, 3c* show interference between the precipitation lines but with a weaker ‘spurlike’ protrusion which extended rectilinearly a short distance from the fused lines = ‘reaction of partial identity’. *Experiment 4* with EaH and PqH (the enzymatically modified EaH) also shows the spur‐phenomenon = ‘reaction of partial identity’. Similar ‘reaction of partial identity’ was obtained if physically modified EaH by heating was used instead of PqH.

**Fig. 3:**
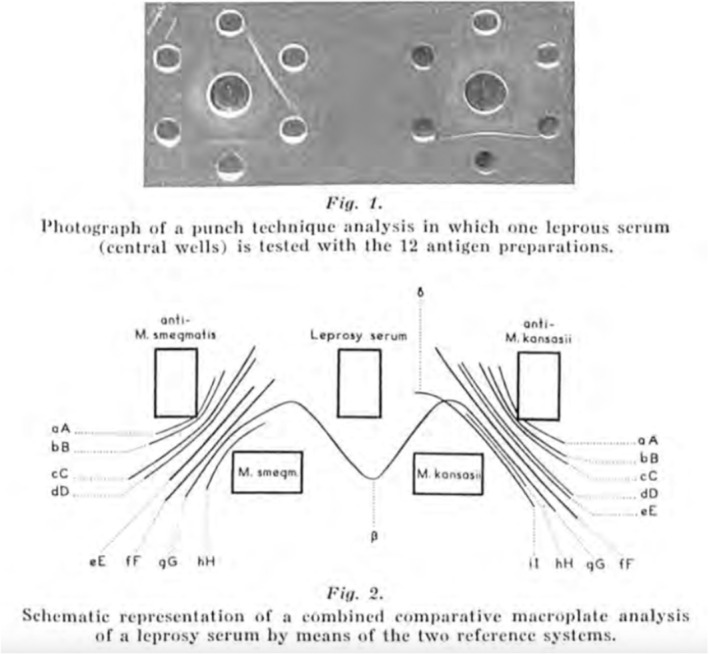
Top is the original Fig. 1 in Reference (8) where one serum from a leprosy patient (the two central wells) and antigens from 12 different mycobacteria were analyzed by gel diffusion technique (*Mycobacterium avium*, *M. balnei*, *M. battaglini*, *M. bovis var. BCG*, *M. fortuitum*, *M. kansasii*, *M. marianum*, *M. microti*, *M. phlei*, *M. smegmatis*, *M. tuberculosis*, and Lepromin). Three precipitates are seen, so the leprosy serum contained only antibodies against one antigen present in three mycobacterial species. Bottom is the original Fig. 2 in Reference (8), and it is a drawing of the results of a gel diffusion experiment using a reference *M. smegmatis* antiserum (left), serum from a leprosy patient (middle) and a reference *M. kansasii* antiserum, antigen preparation from *M. smegmatis* and from *M. kansasii*. The reference antisera were obtained by immunizing sheep (8). Serum from the leprosy patient contained antibodies against one antigen present in anti‐*M. smegmatis* reference antiserum and in anti‐*M. kansasii* reference antiserum and the precipitation line showed ‘reaction of identity’. The designation of the precipitation lines (e.g., aA and bB) between the two reference antisera and the antigens from their corresponding bacteria was described in earlier publications from the authors.

Ouchterlony systematically investigated the rules for formation of the precipitation lines in gel and why ‘reaction of identity’, ‘reaction of partial identity’, and ‘reaction of non‐identity’ could be explained. He published his ground‐braking results in 1953 (7), based on ingenious but simple gel‐diffusions experiments employing egg albumin from hen, egg albumin from the zoologically related guinea hen, egg albumin from the less zoologically related duck, egg albumin from hen modified by treatment with enzyme from *Bacillus subtillis*, and the zoologically non‐related conalbumin (= ovotransferrin) from hen's egg. The results are shown in Fig. 2. These experiments defined ‘reaction of identity’, ‘reaction of partial identity’, and ‘reaction of non‐identity’. The results imply ‘that in multiple antigen‐antibody systems of a precipitate spectrum where each line corresponds to one pair of antigen and antibody, that formation of a precipitate does not disturb the diffusion of such components of the system that are not involved in that particular immunoreaction’. The ‘reaction of partial identity’ will appear, ‘provided that the antiserum contains antibodies of varying specificity’. Ouchterlony later published an article, which demonstrated, that the gel diffusion technique could also be used to analyze the antibody response in leprosy patients to *M. leprae* and other related mycobacteria and even compare it with reference antisera against *M. smegmatis* and *M. kansasii* obtained by immunizing sheep Fig. 3 (8).

## FURTHER DEVELOPMENT OF ANTIGEN–ANTIBODY REACTIONS IN GEL

**Fig. 4:**
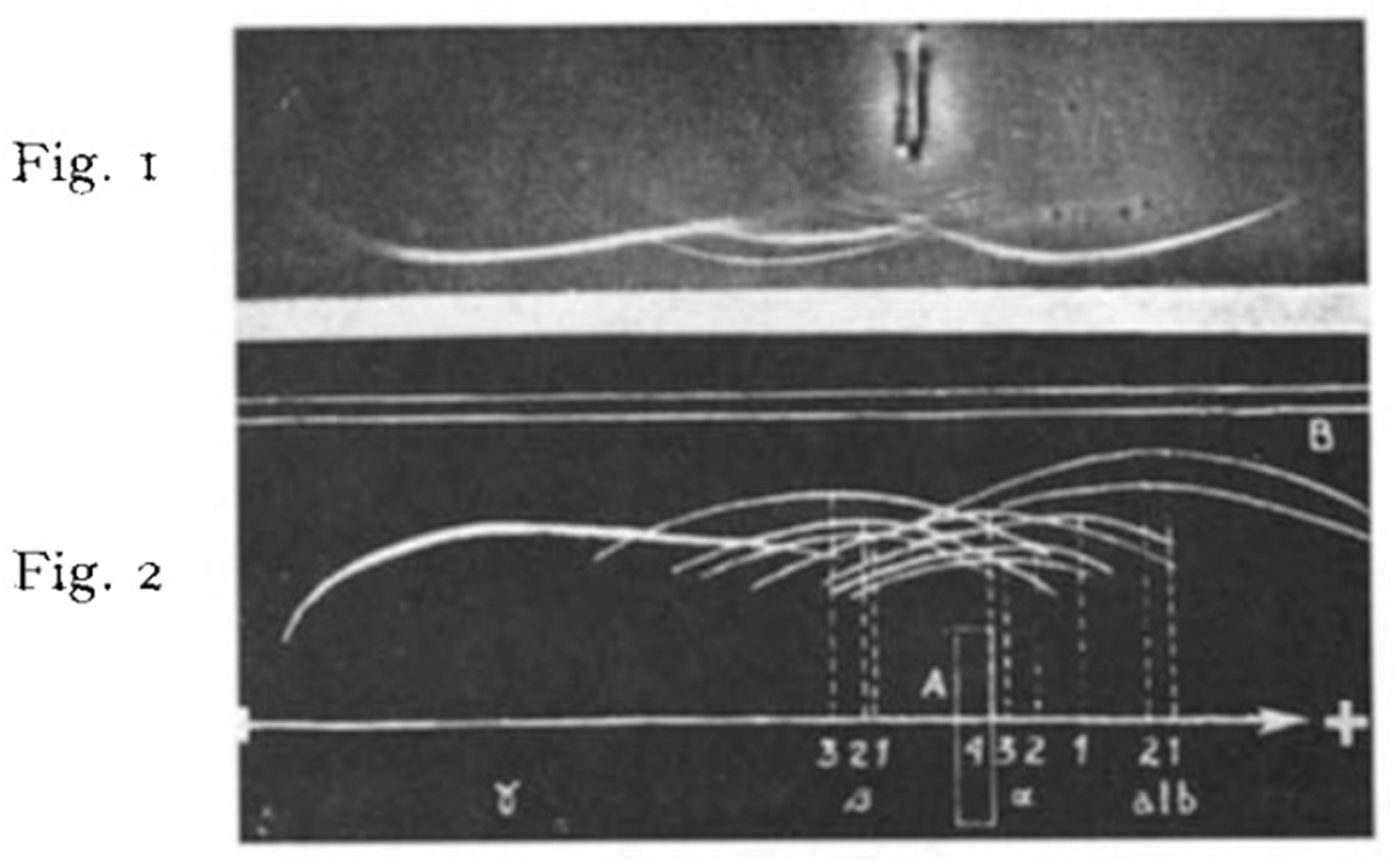
Original Fig. 1 in Reference (9). Immune electrophoresis (pH 8) combined with agar‐gel diffusion of human serum proteins and horse antiserum against human serum. The human serum is placed in the well and separated by electrophoresis, anode (+) to the right. After electrophoresis, horse antiserum is placed in a trench (white) next to the direction of the electrophoresis and the gel diffusion begins and immune precipitates are formed between each human protein and the corresponding antibodies in the horse antiserum. The precipitates are identified in original Fig. 2 by the numbers and dashed lines. In Fig. 1, albumin is the strong precipitate to the right (anode) and IgG is the strong precipitate to the left (cathode).

**Fig. 5:**
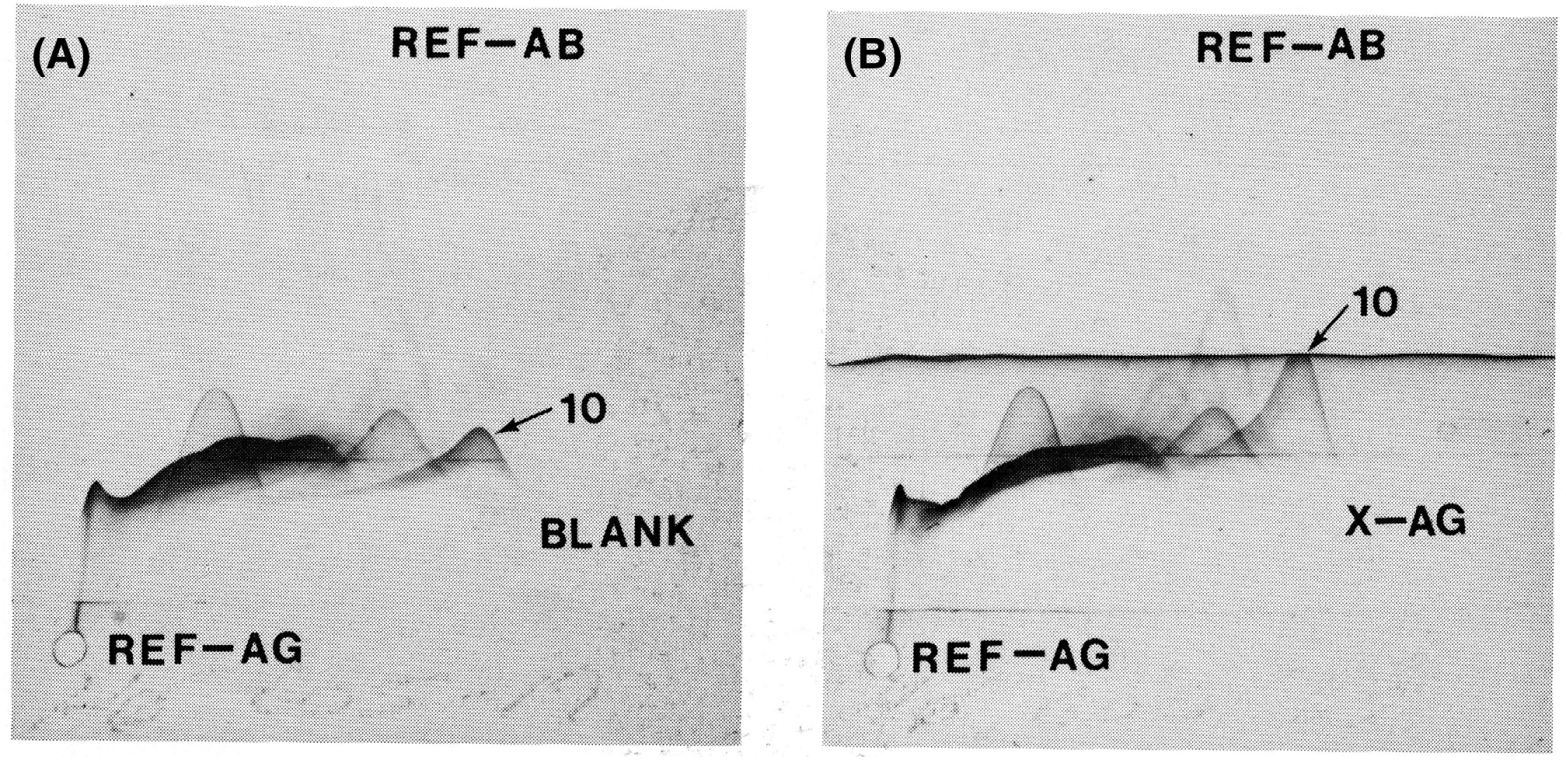
The original Fig. 5 in Reference (19) illustrates the taxonomic use of crossed immunoelectrophoresis. The reference bacterial antigens (REF‐AG) consist of a pool of sonicates of 17 serotypes of *Pseudomonas aeruginosa* (St‐Ag 1–17). The reference antibody (REF‐AB) is a pool of purified and concentrated IgG from sera of 10 rabbits which have been immunized with St‐Ag 1–17. The X‐AG is a sonicate from *H. influenzae* type b. (A, B): The antigens in the REF‐AG are placed in a small well in the agarose gel and separated by electrophoresis (pH 8.6) for 1 h (anode to the right). The gel is then placed on another 5 × 5 cm glass plate and an intermediate gel containing either buffer BLANK (A) or the X‐AG (B) is placed on the glass plate, and the remaining 3/5 of the glass plates is covered with agarose gel containing the REF‐AB. The REF‐AG and the X‐AG are then moved into the antibody‐containing gels by electrophoresis, anode at the top. This variation of crossed immunoelectrophoresis is called ‘crossed‐line immunoelectrophoresis’ since the X‐AG will move as a line, whereas the REF‐AG will move as circles due to the circular shape of the well. (A) shows the stained (Coomassie Brillliant Blue) reference immunoprecipitate pattern of REF‐AG and REF‐AB. One of the precipitates is named antigen 10, and it is the essential GroEL Chaperonin which mediate the ATP‐dependant protein folding (19, 22, 23), and (B) shows the ‘reaction of partial identity’, since IgG which reacts with both REF‐AG and X‐AG has been bound (absorbed) by the X‐AG which forms a linear precipitate. The area of remaining REF‐AG specific IgG precipitate has therefore increased (‘absorption in situ’) (19).

Ouchterlony's publications about gel diffusion influenced colleagues in other countries and Pierre Grabar from France together with his young American student C. A. Williams combined electrophoresis with gel diffusion (immune electrophoresis) in order to separate the antigens so that the precipitation lines were easier to identify (Fig. 4) (9). By this method, the components of a mixture can be defined by two independent criteria – the electrophoretic mobility and the antigenic specificity (9). This method, however, was only semiquantitative but in 1965 Carl‐Bertil Laurell from Lund, Sweden, described the ‘antigen‐antibody crossed immunoelectrophoresis’ (10), and in 1966, he described the ‘quantitative estimation of proteins by electrophoresis in agarose gel containing antibodies’ (11) which was also named rocket immunoelectrophoresis or electroimmunoassay. In the modification of crossed immunoelectrophoresis described by H.G.M Clarke and T. Freeman from UK (12, 13), it was used especially for quantitation of proteins. Further development of quantitative immunoelectrophoretic methods took place in Niels Harboe's Protein Laboratory, University of Copenhagen (14) where a large group of young scientists worked and published doctoral theses, original papers and four books which described the many variants of the technique (15, 16, 17, 18) each of these books became a scientific ‘Bible’ for many other young scientist including the author of this survey (Fig. 5) (19). One of the many advantages of crossed immunoelectrophoresis was that the slow diffusion of proteins in gel was replaced by rapid electrophoretic migration of the proteins at a pH (8.6) where IgG was not migrating and the sensitivity of the method was increased by Coomassie Brilliant Blue‐staining of the immunoprecipitates, so that the results from an experiment which started in the afternoon could be read the next morning (15).

However, it should be mentioned that in a rare case a presumed immunoprecipitate in gel between a bacterial antigen and IgG was not due to a specific immune reaction. This was the case with protein A from *Staphylococcus aureus*, which binds to the Fc part of IgG in serum from all humans and the interpretation of that phenomenon was originally, that all humans produce antibodies against *S. aureus* (20). However, a Swedish scientist from Lund showed that the precipitate was due to the reaction between protein A and the Fc part of IgG (21).

## CONCLUSION

The experiments of Ouchterlony were rather simple but very well planned and meticulously carried out. His conclusions were therefore so convincing that colleagues in other countries rapidly started using and improving his methods and his description in his publications of ‘reaction of identity’, ‘reaction of partial identity’, and ‘reaction of non‐identity’ became classically and his method is still called Ouchterlony double immunodiffusion technique (3, 4, 7, 8).

## CONFLICT OF INTEREST

I have no conflict of interest.

## Data Availability

Data sharing is not applicable to this article as no new data were created or analyzed in this study.
